# Cell-Penetrating Peptides as a Tool for the Cellular Uptake of a Genetically Modified Nitroreductase for use in Directed Enzyme Prodrug Therapy

**DOI:** 10.3390/jfb10040045

**Published:** 2019-10-01

**Authors:** Simon D. Anderson, Robert J. Hobbs, Vanessa V. Gwenin, Patrick Ball, Lindsey A. Bennie, Jonathan A. Coulter, Chris D. Gwenin

**Affiliations:** 1 School of Natural Sciences, Bangor University, Bangor, Gwynedd, Wales LL57 2DG, UK; s.anderson@bangor.ac.uk (S.D.A.); r.j.hobbs@bangor.ac.uk (R.J.H.); v.gwenin@bangor.ac.uk (V.V.G.); chp60c@bangor.ac.uk (P.B.); 2 School of Pharmacy, Queen’s University Belfast, Belfast BT7 1NN, UK; lbennie01@qub.ac.uk (L.A.B.); j.coulter@qub.ac.uk (J.A.C.)

**Keywords:** nitroreductase, cell-penetrating peptide, prodrug therapy, darkfield imaging, nanoparticle

## Abstract

Directed enzyme prodrug therapy (DEPT) involves the delivery of a prodrug-activating enzyme to a solid tumour site, followed by the subsequent activation of an administered prodrug. One of the most studied enzyme–prodrug combinations is the nitroreductase from *Escherichia coli* (NfnB) with the prodrug CB1954 [5-(aziridin-1-yl)-2,4-dinitro-benzamide]. One of the major issues faced by DEPT is the ability to successfully internalize the enzyme into the target cells. NfnB has previously been genetically modified to contain cysteine residues (NfnB-Cys) which bind to gold nanoparticles for a novel DEPT therapy called magnetic nanoparticle directed enzyme prodrug therapy (MNDEPT). One cellular internalisation method is the use of cell-penetrating peptides (CPPs), which aid cellular internalization of cargo. Here the cell-penetrating peptides: HR9 and Pep-1 were tested for their ability to conjugate with NfnB-Cys. The conjugates were further tested for their potential use in MNDEPT, as well as conjugating with the delivery vector intended for use in MNDEPT and tested for the vectors capability to penetrate into cells.

## 1. Introduction

The ability of materials to traverse the cell membrane is an important factor that has to be taken into consideration when devising new medical treatments, requiring careful engineering to aid in target cell uptake [1]. One of the most common forms of cell internalisation for molecular cargoes is endocytosis. This process generally involves the formation of a vesicle surrounding the cargo, which is then engulfed into the cell [2]. However, contents within the vesicle/endosome are often moved into the endo-lysosomal system and are digested by acid hydrolases [3], rendering the cargo unusable for its desired function. While this process is ideal for the uptake of nutrients and for the protection of cells from antigens and toxins [4], it places a restriction on the therapeutic potential of novel therapies. As such, it is important to discover novel approaches to efficiently enable target cell uptake, for which cell-penetrating peptides (CPPs) represent one solution. CPPs are short peptides [5], often no more than 30 amino acids in length [6]. Some CPPs possess the ability to pass through the cell membrane and bypass the endosomal system, leaving cargoes intact [7]. It is often reported that there are several properties that CPPs possess which enable them to efficiently pass into cells including being rich in arginine and/or tryptophan residues [8,9,10] and possessing an overall positive charge at physiological pH (~7.4) [10]. CPPs have already been shown to deliver a wide range of cargo into cells, including nucleic acids [11], proteins [12], and nanoparticles [13].

Directed enzyme prodrug therapy (DEPT) has previously been explored as an emerging form of cancer treatment [14,15,16,17,18,19,20]. DEPT involves the delivery of a prodrug activating enzyme to a solid tumour site, whereby after cell internalisation of the enzyme, a prodrug is then administered. One of the most studied nitroreductase–prodrug combinations is nitroreductase from *Escherichia coli* (NfnB) *K-12*, used in combination with the prodrug; CB1954 (5-(aziridine-1-yl)-2,4-dinitrobenzene). NfnB converts CB1954 into either of the toxic 2’- or 4’-hydroxylamine derivatives, with this combination having had a previous promising clinical outcome [21]. A range of strategies has been investigated to directly deliver the enzyme or the encoding DNA to the target site, including viruses (VDEPT) [22,23], antibodies (ADEPT) [24], peptides [25], and cationic lipids [26]. We have investigated a novel form of DEPT that involves the incorporation of gold-coated superparamagnetic iron oxide nanoparticles (AuMNP) as the delivery vehicle used to direct the enzyme to the tumour site, an approach termed magnetic nanoparticle directed enzyme prodrug therapy (MNDEPT) [14,27]. A genetically modified nitroreductase (NfnB-Cys) is conjugated to the surface of the nanoparticles [14,28] with conjugates already proven to retain their prodrug reducing capability [29]. 

However, it has not yet been established whether the NfnB-Cys enzyme could be conjugated to a CPP to enhance target cell uptake while retaining the reduction activity of CB1954. For these experiments, two CPPs were chosen (HR9 and Pep-1) for conjugation to NfnB-Cys to assess cellular uptake. Both CPPs have been shown to conjugate to peptides and protein structures, improving internalization [30,31,32,33]. It was anticipated that the same improved internalization effect would be seen with NfnB-Cys. The aims of this work were to assess conjugation of the CPPs onto NfnB-Cys, and what effects this had on the enzymes activity and ability to reduce the CB1954 prodrug and cause cell death. Further to this was to conjugate NfnB-Cys and the CPPs onto magnetic nanoparticles intended for use in MNDEPT and again test for the ability for the NfnB-Cys to reduce CB1954 causing cell death.

## 2. Results

### 2.1. NfnB-Cys Expression and Purification

**Figure 1:**
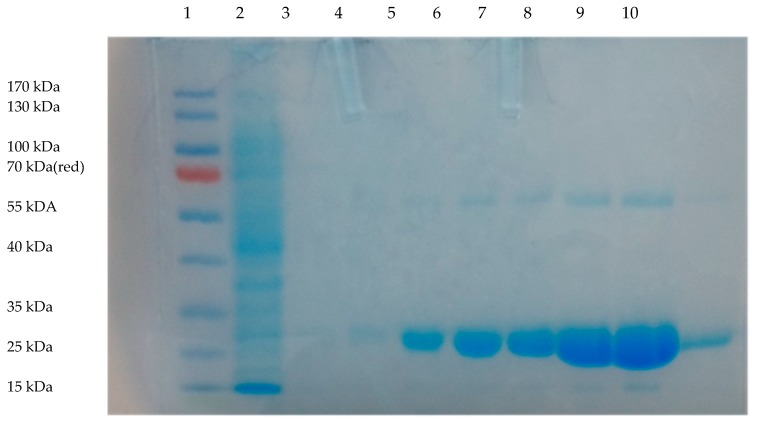
SDS-PAGE gel of the genetically modified nitroreductase (NfnB-Cys) purification using metal ion affinity chromatography (IMAC): lane 1, protein ladder (Thermoscientific PageRuler 15–170 kDa); lane 2, flow-through after applying supernatant to column; lane 3, flow-through after applying 10 mM imidazole to column; lane 4, 50 mM imidazole eluent; lane 5, 100 mM imidazole eluent; lane 6, 200 mM imidazole eluent; lane 7, 300 mM imidazole eluent; lane 8, 500 mM imidazole eluent; lane 9, 800 mM imidazole eluent; lane 10, 1000 mM imidazole eluent.

The NfnB enzyme is native to *E. coli*, but has been genetically modified by our research group to contain six Cys-tags on the N-terminus of the protein [14]. The six Cys-tags aid the in the covalent binding of the NfnB-Cys onto gold nanoparticles [14,28]. The gene was cloned into the pET28a(+) vector, which added an additional six histidine residues after the Cys-tag for downstream purification of the protein using metal ion affinity chromatography (IMAC). The SDS-PAGE gel of the purification is presented in Figure 1, where the NfnB-Cys enzyme, eluted strongly between 300–800 mM imidazole, with the highest concentration being the 500 mM fraction. Due to the high concentration of protein, both monomeric and dimeric units were seen at ~27.3 kDa and 54.6 kDa. The NfnB-Cys enzyme was obtained at a yield of up to 5 mg/mL.

### 2.2. Conjugation of HR9 and Pep-1 to NfnB-Cys

**Figure 2:**
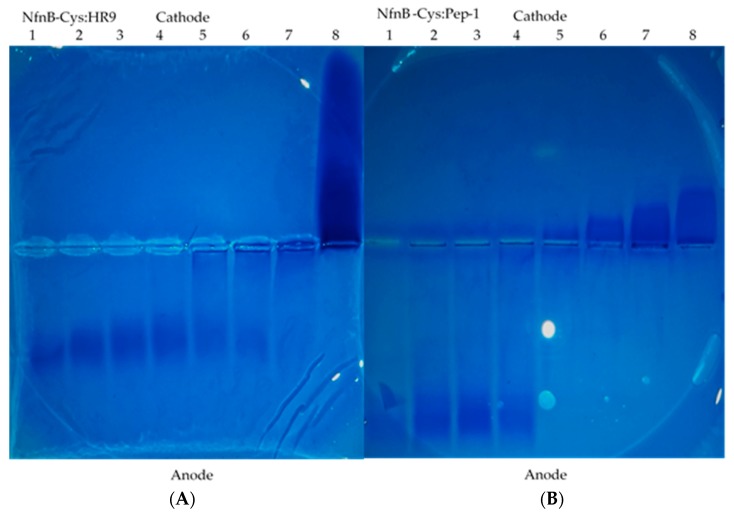
Agarose gels of the NfnB-Cys enzyme conjugated with HR9 (**A**) and Pep-1 (**B**). Gels have unconjugated NfnB-Cys in lane 1, increasing ratios of NfnB-Cys:cell-penetrating peptides (CPPs) in lanes 2, 3, 4, 5, 6, and 7 of 1:0.1, 1:0.2, 1:1, 1:5, 1:10, and 1:15 respectively, and unconjugated CPP in the lane 8.

CPPs can be non-covalently conjugated to a wide range of materials [30,32,33,34,35,36,37,38]. One of the aims of this work was to attempt to improve the cellular uptake of the NfnB-Cys into cells, conjugation of CPPs onto the NfnB-Cys could aid in this process. CPPs were conjugated onto the NfnB-Cys at varying ratios. A further aim is to conjugate the NfnB-Cys onto AuMNPs which act as the delivery vector for our DEPT method, however, it needed to be established if the CPPs could interact with the NfnB-Cys without causing any negative effect. To look for proof of binding of the CPPs HR9 and Pep-1 to NfnB-Cys, native agarose (1%) gels were performed, with images of the stained gels shown in Figure 2 with the free NfnB-Cys, free CPP, and NfnB-Cys:CPP conjugate migration through the gels being analysed. A common feature of many CPPs is their cationic charge, therefore if the CPPs bind to the NfnB-Cys, the charge will differ from that of free NfnB-Cys, with the conjugates differently migrating through the agarose gel. As seen in Figure 2A, HR9 is positively charged, shown by migration towards the anode, whilst free NfnB-Cys is negative, shown by migration towards the cathode. Migration of the NfnB-cys-HR9 conjugates is retarded through the gel resulting in a streaked pattern. Similarly, as seen in Figure 2B, the Pep-1 CPP is also positively charged and migrates to the anode, whilst the NfnB-Cys:Pep-1 conjugates, are again retarded through the gel, particularly at higher Pep-1 ratios (1:5, 1:10, and 1:15). A similar effect can be seen in the 1:15 ratio of NfnB-Cys conjugated with HR9, although it is not as pronounced as the effect seen with Pep-1.

### 2.3. NfnB-Cys and NfnB-Cys:CPP Conjugate Reactivity to CB1954

**Figure 3:**
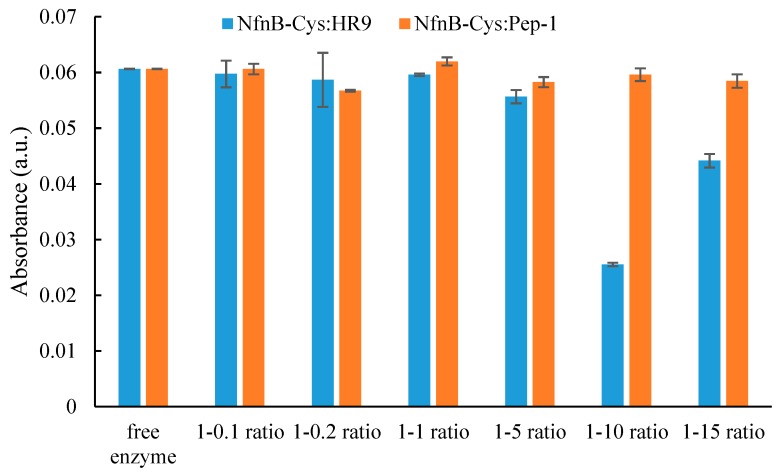
The change in absorbance at 420 nm over 15 min for NfnB-Cys conjugated with varying ratios of HR9 (blue) or Pep-1 (orange) (wt:wt). The reactions were carried out in the presence of NADH (1200 µM) and CB1954 (1000 µM).

The genetically modified NfnB-Cys enzyme reactivity towards the prodrug CB1954 has previously reported by Gwenin et al. [14,39]. The addition of a Cys-tag was shown not to negatively affect enzymatic activity [28], but the conjugation of the CPPs onto the NfnB-Cys may influence enzyme reactivity towards CB1954. For this reason, NfnB-Cys:CPP conjugates were analysed for their CB1954 reactivity and compared relative to the free NfnB-Cys enzyme, the data for which is presented in Figure 3.

When NfnB-Cys was conjugated with HR9 at varying ratios, hydroxylamine product formation could still be seen at 420 nm, however, there appeared to be a significant effect on product formation at ratios above 1:1. The experiment was repeated for NfnB-Cys:Pep-1 conjugates and no major effect on product formation was seen for all ratios tested. Kinetic parameters were then determined by generating a Michaelis–Menten profile for the conjugates (Table 1).

The comparison of the NfnB-Cys:CPP conjugates with our previously reported free NfnB-Cys enzyme kinetics [29] and indicated an alteration in the enzyme’s kinetic behaviour, for both NfnB-Cys:CPP conjugates. The k_cat_ and K_m_ values presented are the apparent (app) values measured at 400 µM NADH. The CPPs decreased efficiency of NfnB-Cys; k_cat_/K_m_ = 0.0026 µM^−1^s^−1^ for NfnB-Cys:HR9 and 0.0033 µM^−1^s^−1^ for NfnB-Cys:Pep-1, compared to k_cat_/K_m_ = 0.011 µM^−1^s^−1^ for NfnB-Cys). The reaction parameters had to be modified to use a higher concentration of NfnB-Cys:HR9/Pep-1 to obtain full kinetic profiles of the conjugates, due to prodrug cost, therefore only the k_cat_/K_m_ constant can be directly compared between the NfnB-Cys enzyme and the NfnB-Cys:CPP conjugates. In comparison the NfnB-Cys:HR9 shows a slightly higher product turnover compared to the NfnB-Cys:Pep-1conjugate (k_cat_(app) = 9.1 s^−1^ and 8.4 s^−1^ respectively), however, the NfnB-Cys:Pep-1 demonstrates a higher affinity than NfnB-Cys:HR9 for the CB1954 (K_m_(app) = 2400 µM and 3400 µM respectively). These results show that when conjugated to the CPPs; HR9 and Pep-1 at a 1:1 ratio NfnB-Cys has a 65–74% decrease in kinetic efficiency. These results are presented in Table 1.

### 2.4. HPLC Profiles of NfnB-Cys and NfnB-Cyse:CPP Reaction Products

Previously we have shown that after a 30 min reaction time, NfnB-Cys reduced CB1954 to two products (namely 2-NHOH and 4-NHOH) at a ratio of 32:68 respectively [29]. Following the same procedure, conjugates of NfnB-Cys and the CPPs at a 1:1 molar ratio were tested for their ability to reduce CB1954. Reaction product ratios were established, with the 4-NHOH product eluting at approximately 5 min and the 2-NHOH eluting at 10 min [29,39]. The product ratios obtained for the reactions between NfnB-Cys:CPP conjugates and CB1954 are presented in Table 2; the NfnB-Cys:HR9 produced products at a ratio of 36:64 (2’-NHOH vs 4’-NHOH), whilst the NfnB-Cys:Pep-1 produces the 2-NHOH and 4-NHOH at a ratio of 35:65.

### 2.5. Effect of NfnB-Cys and NfnB-Cys:CPP Conjugate on Cell Viability

**Figure 4:**
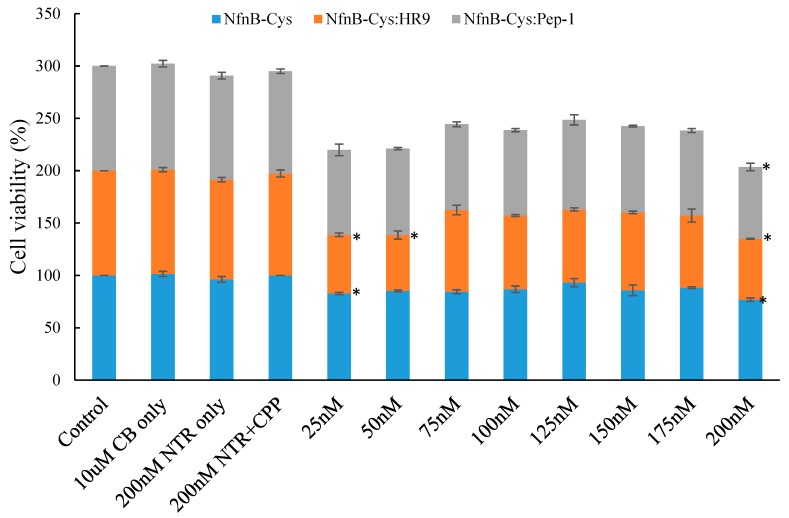
The percentage cell survival of SK-OV-3 cells after 4 h incubation with: cell culture medium only, 10 µM CB1954 only, 200 nM NfnB-Cys only, 200 nM NfnB-Cys:CPP only, and increasing concentrations of either; NfnB-Cys (blue), NfnB-Cys:HR9 (orange), or NfnB-Cys:Pep-1 (grey) (25–200 nM) in the absence of NADH. Data points determined to be statistically significant by Dunnett test are marked with a *. All data points represent at least three repeats and error bars indicate ± 1 standard deviation.

Cell viability of SK-OV-3 cells presented as percentage cell survival was determined using an increasing concentration of NfnB-Cys and NfnB-Cys:CPP conjugate and a constant CB1954 concentration (10 µM). Cells were incubated with medium, prodrug, and enzyme separately as controls. NfnB-Cys was tested for its ability to induce cell death by the reduction of CB1954. This combination showed an average cell viability of 80% at an NfnB-Cys treatment concentration of 200 nM, with the full results shown in Figure 4. Next, the NfnB-Cys:HR9 and NfnB-Cys:Pep-1 conjugates were also tested for their ability to cause cell death, and compared relative to free NfnB-Cys, again full results are displayed in Figure 4. 

At each concentration tested, the NfnB-Cys:HR9 conjugates (25–200 nM) appear to be more effective at causing cell death compared to NfnB-Cys alone (on average by 15%). Neither Pep-1 nor HR9 CPPs conferred any direct cytotoxicity towards SK-OV-3 cells. These results suggest that NfnB-Cys conjugates exhibited a greater endocytotic potential, because, as shown from the kinetic experiments, cell kill could not be attributed to enhanced enzyme efficiency. The data were analysed for statistical significance by F-test with all data sets demonstrating levels of statistical significance (*P* < 0.005), with the individual data points being analysed using the Dunnett test. Data points marked with a * exceeded the Dunnett critical value indicating statistical significance.

### 2.6. Effect of AuMNPs and AuMNP Conjugates on Cell Viability

**Figure 5:**
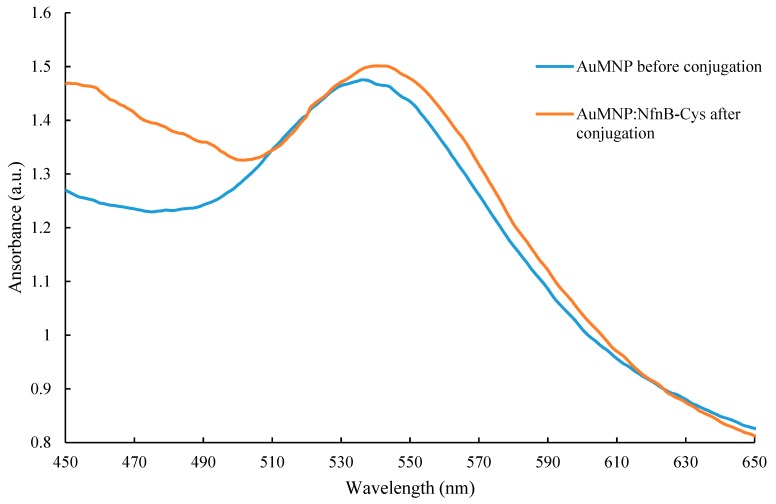
Full-spectrum (450–650 nm) UV-vis spectrum of gold-coated superparamagnetic iron oxide nanoparticles (AuMNPs) before (blue) and after (orange) conjugation with NfnB-Cys at a ratio of 1:270 of AuMNP:NfnB-Cys. Scans were taken 48 h apart.

NfnB-Cys has been shown to successfully conjugate onto AuNPs [14], therefore it was considered highly probable the same would be observed when conjugating onto AuMNPs. Conjugation of NfnB-Cys onto AuMNPs was assessed by UV-Vis, Figure 5 is the overlay of UV-Vis scans between 450 and 650 nm. Here it is observed that post conjugation the λ-max of the gold peak has increased by 4 nm from 536 to 540 nm, an indication of successful conjugation. 

**Figure 6:**
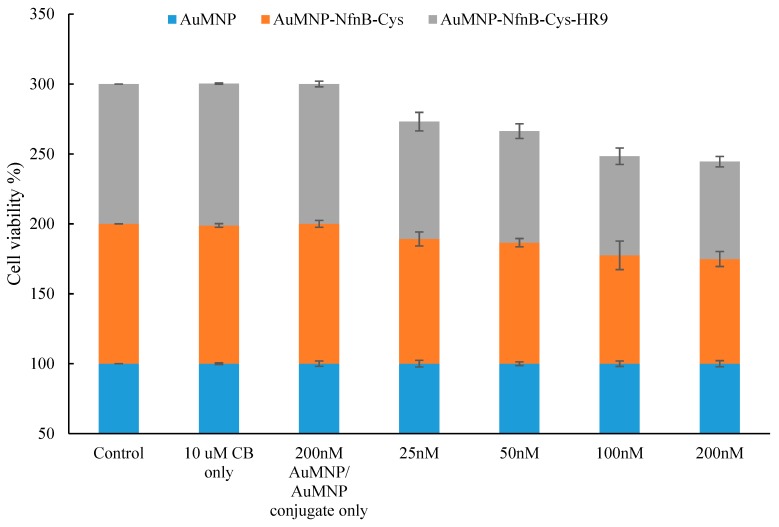
The percentage cell survival of SK-OV-3 cells after 4 h incubation with: cell culture medium only, 10 µM CB1954 only, 200 nM AuMNP only, 200 nM AuMNP:NfnB-Cys only, or 200 nM AuMNP:NfnB-Cys:CPP only as control wells. Reaction wells contained increasing concentrations of either; AuMNP (blue), AuMNP:NfnB-Cys (orange), or AuMNP:NfnB-Cys:HR9 (grey) (25–200 nM) in the absence of NADH. Complete reactions also contain CB1954 at a 10 µM concentration. All data points represent at least three repeats and error bars indicate ± 1 standard deviation.

There was a concern that, while performing the MTT assay on the AuMNPs, any exposed iron nanoparticles would cause excess oxidation of the MTT yielding a bias on the final cell viability percentage [40,41]. A brief experiment was performed to assess if the AuMNPs would cause excess oxidation of the MTT causing a result bias. The AuMNPs caused a large excess of oxidation of the MTT indicating a different cell culture assay would be required (data not shown). Due to this, the calcein assay was selected as it requires the use of cellular esterase’s to convert calcein-AM into the fluorescent calcein, an initial test showed the AuMNPs are not able to reduce calcein-AM to calcein indicating the assay could be used without the risk of an experimental bias (data not shown). Figure 6 is the cell viability results of cells treated with: AuMNPs, AuMNP:NfnB-Cys, or AuMNP:NfnB-Cys:HR9, here the range of concentrations examined are the same as the cell viability experiments not containing AuMNPs that are described in Section 2.5, Section 3 and Section 4.7.

The AuMNPs do not demonstrate any direct toxicity towards the SK-OV-3 cells. As expected, when AuMNP:NfnB-Cys and AuMNP:NfnB-Cys:HR9 conjugates were treated onto cells, there was cell kill, which was taken to be the NfnB-Cys reducing the CB1954 due to the lack of toxicity presented in the conjugated control samples. Here once again the conjugates with the HR9 do present a slightly better cell kill overall than the AuMNP:NfnB-Cys, however the increase in the cell kill is minimal at best. The data were analysed for statistical significance by F-test with all data sets demonstrating levels of statistical significance (*P* < 0.005). The Dunnett test could not be performed to determine individual data points statistical significance due to the low number of concentrations tested.

### 2.7. Darkfield Imaging

**Figure 7:**
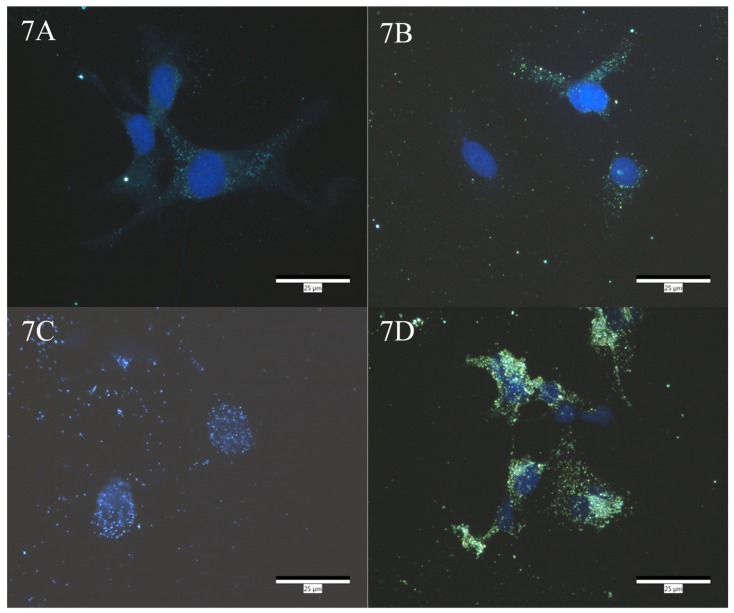
Enhanced darkfield imaging of cells treated with either: Dulbecco’s modified eagle medium (DMEM), AuMNP, AuMNP:NfnB-Cys, or AuMNP:NfnB-Cys:HR9, with the images for each presented in (**A**), (**B**), (**C**), and (**D**) respectively. The cells were treated with DAPI as a co-stain for imaging of the cell nucleus. The scale bar is 25 µm.

Enhanced darkfield imaging was performed on SK-OV-3 cells treated with either: Dulbecco’s modified eagle medium (DMEM), AuMNP, AuMNP:NfnB-Cys, or AuMNP:NfnB-Cys:HR9, with the HR9 at a 1:1 ratio with the AuMNP. Treatments were done to assess cell uptake of the nanoparticle/nanoparticle conjugates and if the addition of the HR9 aided in increasing cellular uptake. On the basis that the HR9 conjugates appeared to be superior in cell culture testing as an isolated conjugate, only the NfnB-Cys:HR9 combination was progressed to AuMNP testing. Figure 7 is the enhanced darkfield imaging of these slides, Figure 7A is the imaging of cells treated with just DMEM to act as a control, with the cell nucleus stained blue with DAPI. The untreated control cell (panel A) acts as a negative in relation to AuNP internalisation, to which any changes in terms of particle intensity are compared following treatment with AuMNPs. Figure 7B–D are images taken of cells treated with AuMNP, AuMNP:NfnB-Cys, or AuMNP:NfnB-Cys: HR9 respectively, again the cell nuclei are counterstained blue with DAPI. These images have a higher frequency of intense areas within the cells, which are absent when compared to control cells, potentially representing the presence of AuMNPs within the cells. The increased bright areas within the cells in the images indicate that the AuMNPs have internalized into the SK-OV-3 cells and when conjugated with NfnB-Cys, has an increase in internalized nanoparticles. However, the AuMNP has a significant enhancement in nanoparticle internalization when conjugated with a CPP, shown in Figure 7D.

## 3. Discussion

Since their initial discovery in 1988, CPPs have presented as a unique tool for aiding in the uptake and delivery of a range of cargoes for medical applications. Literature shows that CPPs bind non-covalently to specific cargo [36,42,43,44], the initial work was to determine whether HR9 and Pep-1 could also non-covalently conjugate with our enzyme as a majority of other studies focus on conjugation with inorganic substances such as quantum dots. Conjugation success was confirmed, showing that both HR9 and Pep-1 conjugates significantly alter the electrophoretic migration of NfnB-Cys through the gel (Figure 2A,B) [45]. The HR9 peptide had a greater influence on NfnB-Cys migration, compared to Pep-1, possibly a result of possessing a greater positive charge, caused by the large number of cationic arginine residues [44], nine of which are found in the HR9 CPP [36,46], compared with the one arginine residue found within the Pep-1 [46].

The high positive charges that CPPs possess could present a possible problem in terms of interactions with the physical structure of proteins, this becomes a potential issue for enzyme conjugation as it has previously been established that changes to the physical structure of the NfnB enzyme can cause a change in the product ratio formed when the enzyme is reacted with the CB1954 prodrug [28]. Analysis by HPLC of the product ratio formed for the NfnB-Cys:CPP conjugates showed little to no change from the free NfnB-Cys enzyme (ratio 32:68). NfnB-Cys:HR9 produced a ratio of 36:64, whilst NfnB-Cys:Pep-1 produced a ratio of 35:65. The fact that the CPPs do not heavily influence the product ratio formed here indicates that the CPPs are not causing any major alteration to the physical structure of the NfnB-Cys enzyme when they conjugate with it. This is important as it means that the enzyme can still convert the CB1954 into its pharmaceutically active form without CPP interference. A concern that would limit the efficacy of this treatment is that the CPPs are non-covalently bound with the AuMNP:NfnB-Cys conjugate, which could dissociate when introduced to a patient. Stability studies were carried out on AuMNP:NfnB-Cys:CPP conjugates (data not shown) in the cell culture media used in the experiments discussed in this paper. The conjugates were left at 37 °C to simulate physiological conditions and were stable at up to and including 120 h which would be a semi-realistic representation of treatment time.

As seen from the HPLC experiment, conjugated CPPs had no real effect on the product ratio of NfnB-Cys, however, establishing if CPP conjugation caused an alteration in enzyme kinetics was essential before progressing. As mentioned in Section 2.3, only the efficiency constant (k_cat_/K_m_ µM^−1^s^−1^) can be directly compared between the free and conjugated NfnB-Cys. When looking at the K_cat_/K_m_ of the free NfnB-Cys, a value of 0.018 µM^−1^s^−1^ has previously been reported [29]. Both the k_cat_/K_m_ values for the NfnB-Cys:CPP conjugates show a significant drop in their efficiency, with NfnB-Cys:HR9 having a value of 0.0026 µM^−1^s^−1^ which is 4-fold less than NfnB-Cys and NfnB-Cys:Pep-1 having a k_cat_/K_m_ of 0.0035 µM^−1^s^−1^, a value almost 3-fold lower than NfnB-Cys. Therefore, while the earlier experiments showed very little change in both product ratio and product formation, the efficiency of both conjugates and product turnover has dramatically reduced compared to free NfnB-Cys. It is likely that the change in enzymatic efficacy is not detected in the earlier experiments as UV-Vis absorbance measurements were collected at 15 min, and the HPLC reaction time point was 30 min. However, enzymatic kinetic experiments captured a short time frame, calculated over a 20 s time period. It is likely that whilst the shorter kinetic experiments represent an accurate kinetic picture of the enzyme, the longer experiments possibly indicate a more realistic scenario of the final concentration of products produced as it allows for full reduction and consumption of prodrug. This drastic change in efficiency of the conjugate compared to the free NfnB-Cys does raise a question that requires further research to understand: If the HPLC indicates that the ratio of products formed does not largely differ from the ratio formed by the free NfnB-Cys why does the kinetic data show such a difference? This might indicate that the CPPs have some sort of interaction with the active site of the NfnB-Cys, it may be that there is some slight blockage of the active site, or the CPPs cause a delay in the release of the products from the active site. Further experiments are needed to answer this anomaly in the data.

When examining the NfnB-Cys:CPP conjugates in cell viability assays, there is an observable improvement in the ability of the NfnB-Cys:CPP conjugates to induce cell death over free NfnB-Cys. No additional NAD(P)H was added during the experiments, this was done so that the only cofactor available for the enzyme would be found intracellularly within the SK-OV-3, meaning that the enzyme has to be internalized along with the CB1954 to reduce the prodrug. This increased cell death, and indicates that the addition of CPPs onto the enzyme aids the uptake of the enzyme, allowing the increased cell kill despite the lowered enzymatic efficiency observed. The lower concentration range of NfnB-Cys:HR9 tested in cell culture presents a low cell viability, this can be potentially attributed to the hormetic effect [47], and may indicate a treatment concentration of 25 nM NfnB-Cys:HR9 as a better option compared to the 200 nM treatment concentration.

The NfnB-Cys is intended for use in our patented MNDEPT [27], so in order to assess cellular uptake of the enzyme, it was covalently conjugated to the AuMNPs and assessed for its viability as a delivery vector, further conjugating HR9 with the AuMNP:NfnB-Cys and measuring the ability to cause cell death. The cell viability results of the AuMNP trials presented in Figure 6 indicate that the AuMNPs and the AuMNP conjugates do not themselves have an adverse toxic effect on the SK-OV-3 cells, from this it can be reasoned that any cell kill present is from the AuMNPs/AuMNP conjugates that are able to internalize into the cells along with CB1954. Both of the conjugates tested were able to reduce CB1954, shown by the decrease in cell viability which became more pronounced with the increasing concentration dose. 

At each concentration tested, the conjugate with HR9 attached does show an increase in the cell kill. However, this increase is very small, at a maximum 5–10%. This may indicate that either the ratio of HR9 used for the treatment needs to be increased to achieve a higher internalization rate of conjugates. It can also indicate that HR9 is not able to effectively internalize the AuMNP, meaning a different CPP might present as a better option. It is also possible that all the cell culture data has varying degrees of endosomal trapping, preventing the enzyme from fully reducing the prodrug within the cells, in which case the CPPs may need slight modification to achieve endosomal escape either with the use of an endosomolytic agent or a way to reverse high-affinity binding to cell receptors [48]. Furthermore, uptake efficiency of CPPs into cells can vary based on cell type, here the cell line SK-OV-3 showed CPP uptake, however it must be considered that other cell types may show much more limited uptake or no uptake at all. Along with this further work to be done would involve examining the potential lysosomal trapping and to assess colocalization of the nanoparticle conjugates within the cellular structures.

Darkfield imaging was performed on the three nanoparticle/nanoparticle conjugates to assess if the conjugation of either the NfnB-Cys and/or the HR9 increases the cellular internalization of the nanoparticle. Darkfield imaging allows us to visually inspect cells, and view nanoparticles that are associated within the cell, here imaging was performed in an attempt to assess what observable change, if any, the addition of a CPP onto the conjugate had on the cellular internalization of the conjugate. It is observable that the addition of the NfnB-Cys onto the AuMNP causes more nanoparticles to internalize into the cells, compared with unconjugated AuMNP. This is important as it shows that even without the CPP, the AuMNP:NfnB-Cys conjugate can penetrate into the cells, reinforcing that AuMNPs are a viable choice for use in MNDEPT. The more notable difference is observed with the incorporation of a CPP onto the conjugate. This addition allows a drastically larger number of conjugates to internalize into the cells, indicating that the CPP is being successful in its role.

In conclusion, two different CPPs have been successfully conjugated to the genetically modified NfnB-Cys enzyme at a 1:1 ratio. The HPLC reaction profiles of the NfnB-Cys:CPP conjugates have been described, showing a slight change from the ‘free’ NfnB-Cys enzyme’s product ratio. Kinetic profiles have been established for the conjugates at a 1:1 ratio showing a large drop in the conjugate’s kinetic efficiency. However, when the conjugates are tested in cell viability assays an increased cell kill is observed, consistent with what would be expected with an increased uptake of the enzyme, whilst the CPPs themselves show no observable toxicity. Cell viability assays were also performed on AuMNP, AuMNP:NfnB-Cys, and AuMNP:NfnB-Cys:HR9 conjugates, and the AuMNPs themselves had no observable toxicity towards the cells whilst the other tested conjugates demonstrated an increasing cell kill with increasing treatment concentration. Finally, darkfield imaging presents an increased internalization of AuMNP conjugated when HR9 is also conjugated with the AuMNP:NfnB-Cys, which is consistent with the increased cell kill seen on the AuMNP:NfnB-Cys:HR9 conjugate.

## 4. Materials and Methods

All chemicals were supplied from Fisher Scientific, UK and Sigma Aldrich, UK unless stated otherwise.

### 4.1. Expression and Purification

An *nfnb-cys* gene that had been previously cloned into the pET28a(+) (Novagen, Merck, UK) expression vector [14], was transformed into an *E.coli Rosetta* strain B21 DE3 (Novagen, Merck, UK) and the expression and purification of the NfnB-Cys enzyme was carried out as previously described [14]. Briefly, a colony of *E. coli Rosetta* containing the expression vector (previously confirmed using gel electrophoresis, data not shown) with the *nfnb-cys* gene was added to a Luria-Bertani (LB) inoculant tube (5 mL) also containing kanamycin (50 µg/mL). This was vortexed at 1500 rpm overnight for 16 h. Following this the inoculant was added to a flask containing LB expression medium (500 mL) and kanamycin (50 µg/mL). The bacterial medium was left to grow to an optical density of 0.6–0.7 measured at 590 nm, after which expression of protein was induced by the addition of isopropyl-*β*-_D_-thio-galactoside (IPTG) (2 mL, 100 mM). After 4 h of NfnB-Cys expression, the culture was centrifuged (9318 rcf, 10 min, 4 °C) and cell contents released by firstly suspending the bacterial pellet in imidazole (10 mM, pH 7.2) and then sonicated on ice for 2 min at 40% amplitude, using 30 s bursts. Cell debris was removed by high-speed centrifugation (44,800 rcf, 1 h) and the yellow supernatant passed through a metal ion affinity chromatography column using Ni^2+^, with imidazole as the eluent. NfnB-Cys protein eluted as yellow fractions that were collected and incubated with flavin mononucleotide (FMN) on ice for 1 h to ensure cofactor saturation. The saturated solution was then subjected to PD10 size exclusion chromatography (SEC) to remove both impurities and any residual imidazole eluent, before the NfnB-Cys enzyme was collected in phosphate buffer (50 mM, pH 7.2). The molecular weight and purity of the protein fractions were assessed using 12% SDS-PAGE and visualized using Coomassie blue stain. The concentration of the protein was established using the Bradford assay using a standard BSA curve, according to the manufacturer’s instructions.

### 4.2. CPP Conjugation to NfnB-Cys

Conjugation of the CPPs, HR9 (*CHHHHHHRRRRRRRRRHHHHHHC*) and Pep-1 (*KETWWETWWTEWSQPKKKRKV*), to the NfnB-Cys enzyme was performed as described for conjugation onto quantum dots (*QD*) by Liu et al. [36], and replacing the QD with our NfnB-Cys. This was done by mixing the enzyme and CPP in a silinated Eppendorf at various wt:wt ratios (1:0.1, 1:0.2, 1:1, 1:5 1:10, 1:15). The enzyme: CPP mix was then incubated at 37 °C for 30 min and assessed for conjugation via agarose gel electrophoresis.

### 4.3. Confirmation of CPP Conjugation to NfnB-Cys

A 1% (w/v) agarose gel was prepared by dissolving agarose (1 g) in 100 mL TBE buffer (Tris-HCl; 54 g, Boric acid; 27.5 g, EDTA; 20 mL, 500 mM dissolved in H_2_O 1 L, pH 8) [14]. The gel was then submerged in 1× TBE running buffer and the comb removed. NfnB-Cys:CPP conjugate (20 µL) was mixed with 20 µL of a 2× loading dye (10 mM Tris-HCl pH 6.8, 2% SDS, 0.01% Bromophenol blue, 20% glycerol) [45,49] and loaded onto the gel. Unconjugated NfnB-Cys and CPP were used as controls. The gel was then run at 100 V for 1 h, and visualized using Coomassie blue stain.

### 4.4. NfnB-Cys and NfnB-Cys:CPP Conjugate Activity to CB1954

NfnB-Cys:CPP conjugates and unconjugated NfnB-Cys were assessed for their reactivity to CB1954 by incubating NfnB-Cys (25 µg/mL) or NfnB-Cys:CPP (25 µg/mL, with volume added adjusted for additional CPP in the mixture to ensure 25 mg/mL of NfnB-Cys) with NADH (300 µM), phosphate buffer (*PB*) (50 mM, pH 7.2), and CB1954 (100 µM). Absorbance spectra (200–800 nm) were measured every 90 s for 15 min on a Jasco V-550, UV-vis spectrophotometer. Standard control scans were also run on the NADH, enzyme, enzyme:CPP conjugate, prodrug, and CPP with NADH and CB1954 to ensure the CPPs could not reduce the prodrug.

### 4.5. CB1954 Kinetics

CB1954 kinetic experiments were all carried out in a 96-well microtiter plate (Corning, USA) using a Thermoscientific Varioskan 96-well plate microplate reader [29]. Product formation at 420 nm was measured over time in order to determine the Michaelis–Menten kinetic parameters of CB1954 against the NfnB-Cys:CPP conjugate. CB1954 (0.1–5 mM), NADH (400 µM) and PB (50 mM, pH 7.2) were combined and incubated at 37 °C for 3 min before purified NfnB-Cys or NfnB-Cys:CPP (1:1 ratio) was added (50 μg/mL; again NfnB-Cys:CPP volume added was adjusted to ensure 50 μg/mL of NfnB-Cys was added). Dimethyl sulfoxide (DMSO) solvent concentration was kept constant at 5% v/v to account for any negative solvent related effect [50]. Hydroxylamine yield per second was determined by calculating the change in absorbance over 20 s and the molar extinction coefficient, which is the same for both products (ε = 1200 M^−1^cm^−1^ at 420 nm) [14,18,39,50,51,52,53]. Data gathered was transferred to SigmaPlot 12 (SPSS, Systat Software Inc.) where a non-linear regression tool was used to generate a Michaelis–Menten hyperbolic curve and a report containing the kinetic information of the system.

### 4.6. HPLC

For HPLC analysis, a Dionex Ultimate 3000 HPLC machine Thermoscientific, USA was used with a C18 column (Waters Spherisorb® 5 µm ODS2 4.6 mm × 250 mm C18 column, UK). Experiments were carried out at the following parameters: 50 µL injection volume, 25 °C column oven temperature, UV detection wavelength of 420 nm, and a run time of 30 min [41,42].

HPLC samples were prepared as previously described [29,39]. Briefly, samples were prepared in a 15 mL amber falcon tube (due to the light-sensitive nature of some of the reaction constituents) as follows: NADH (120 µL, 10 mM) NfnB-Cys/NfnB-Cys:CPP (116 µg/mL, volume adjusted), CB1954 (20 µL, 50 mM) then made to a final volume of 1080 µL using PB (50 mM, pH 7.2). This mixture was incubated at 25 °C for 15 min, and then degassed under nitrogen (g) for 15 min, giving a total reaction time of 30 min. Next, 750 µL of the final de-gassed reaction was transferred to a chromacol select 2 mL vial (2-SVW8-CPK) and placed into the HPLC machine. The solvent mixture was acetonitrile/water at a 10:90 ratio, with the acetonitrile increasing at 1% per min. After 20 min, the acetonitrile concentration was altered to keep increasing by 40% per minute which reached a concentration of 100% acetonitrile after 22 min. Eluents were scanned at 420 nm with product peaks being identified against reagent standards carried out using the same protocol: CB1954 (20 µL, 50 mM), NADH (120 µL, 10 mM), and NfnB-Cys (116 µg/mL, volume adjusted for concentration of NfnB-Cys from purification). Ratios of the 2’ and 4’-hydroxylamine products were determined at 420 nm, where both products have equal absorbance [18]. 

### 4.7. Cell Viability Assays

Cell viability assays were performed as previously described [30]. SK-OV-3 (ECACC 91091004) cells were seeded into a 96-well plate (Corning, USA) at a density of 1 × 10^3^ cells per well, in 100 µL Dulbecco’s modified eagle medium (DMEM) containing 10% FBS, 1% L-glutamine, and 1% penicillin-streptomycin and allowed to attach to the plate overnight in a CO_2_ (5%) incubator at 37 °C. After 16 h, the media was carefully aspirated off and fresh media containing increasing concentrations of 25 to 200 nM of NfnB-Cys or NfnB-Cys:CPP conjugate (50 µL) was added to the wells along with CB1954 (100 µM). Wells where only NfnB-Cys, NfnB-Cys:CPP conjugate (200 nM), CB1954 (10 µM) or DMEM (100 µL) were added served as controls. After a 4 h incubation in a CO_2_ (5%) incubator at 37 °C, the treatment media was carefully aspirated off and fresh media (100 µL) added. Cells were then left for 48 h in a CO_2_ (5%) incubator at 37 °C, after which MTT (3-(4,5-dimethylthiazol-2-yl)-2,5-diphenyltetrazolium bromide) (20 µL, 5 mg/mL) was added and then left for a further for 4 h at 37 °C. Culture media was aspirated off and DMSO (100 µL) used to dissolve the purple formazan crystals. Finally, absorbance was read at 570 nm using a Thermoscientific Varioskan Flash plate reader.

### 4.8. Preparation of AuMNP:NfnB-Cys and AuMNP:NfnB-Cys:HR9 Conjugates for Cell Culture and Darkfield Imaging

Previously synthesised AuMNPs [54] were conjugated with NfnB-Cys following the published method for conjugating NfnB-Cys onto gold nanoparticles [14]. Briefly, magnetically purified, 50 nm AuMNPs suspended in sodium citrate dehydrate (1 mM, pH 7.4) were incubated with a volume of NfnB-Cys at a ratio of 1 AuMNP:270 NfnB-Cys to achieve a monolayer coating of the nanoparticles. The volume of NfnB-Cys incubated with AuMNPs was determined based upon the concentration of NfnB-Cys determined by the Bradford assay and the concentration and size of AuMNPs as determined by UV-Vis [14]. Nano-conjugates were left to form at 4 °C for 48 h. A full wavelength UV-Vis scan (450–650 nm) was performed on AuMNPs before and after conjugation, with a change in the gold peak being examined for. A redshift of 3–5 nm of the λ-max of the gold peak indicates successful conjugation [14,55]. AuMNP:NfnB-Cys:HR9 conjugates were prepared by incubating the AuMNP:NfnB-Cys with HR9 at a 1:1 ratio of HR9 to AuMNP, at 37 °C for 30 min.

### 4.9. AuMNP Cell Viability Assays

The calcein assay was performed with AuMNPs and AuMNP conjugates. SK-OV-3 (ECACC 91091004) cells were seeded into a 96-well plate (Corning, USA) at a density of 1 × 10^3^ cells per well, in 100 µL Dulbecco’s modified eagle medium (DMEM) containing 10% FBS, 1% L-glutamine, and 1% penicillin-streptomycin and allowed to attach to the plate overnight in a CO_2_ (5%) incubator at 37 °C. After 16 h, the media was carefully aspirated off and fresh media containing increasing concentrations of 25 to 200 nM of AuMNPs, AuMNP:NfnB-Cys, or AuMNP:NfnB-Cys:HR9 (50 µL) was added to the wells along with CB1954 (100 µM). Wells where only AuMNP, AuMNP:NfnB-Cys AuMNP:NfnB-Cys:CPP conjugate (200 nM), CB1954 (10 µM), or DMEM (100 µL) were added served as controls. After a 4 h incubation in a CO_2_ (5%) incubator at 37 °C, the treatment media was carefully aspirated off and fresh media (100 µL) added. Cells were then left for 48 h in a CO_2_ (5%) incubator at 37 °C, after which the media was carefully aspirated off and 1× calcein DW buffer (100 µL) was added to each well. This was again carefully aspirated off after which 1× calcein DW buffer (50 µL) and 2× calcein AM (50 µL) were added to each well. The plate was then incubated for 30 min in a CO_2_ (5%) incubator at 37 °C. The fluorescence of the sample was measured using an excitation/emission filter of 495/515 nm using a Thermoscientific Varioskan Flash plate reader.

### 4.10. Darkfield Imaging

Darkfield imaging was performed using CytoViva enhanced darkfield imaging. SK-OV-3 cells were seeded onto 8-well Thermo Scientific™ Nunc™ Lab-Tek™ II Chamber Slide&trade glass slides at a density of 1 × 10^4^ cells per well. Sub-confluent cells (~80%) were treated with either DMEM media as a control, unconjugated/‘naked’ AuMNPs, AuMNP conjugated with NfnB-Cys at a ratio of 1:270 of AuMNP:NfnB-Cys, or AuMNP:NfnB-Cys conjugated with HR9, with the CPP conjugated at a ratio of 1:1 with the AuMNP:NfnB-Cys conjugates. Cells were incubated with complexes for 4 h before the treatment media was removed and cells washed twice with DPBS, removing loosely associated external nanoparticles [56,57,58,59,60]. Cells were then fixed using 3.7% formaldehyde and mounted using Vectashield containing 4′,6-diamidino-2-phenylindole (DAPI) (Vector labs), counterstaining the nucleus.

## Figures and Tables

**Table 1:** Michaelis–Menten kinetic data obtained for NfnB-Cys and the conjugated NfnB-Cys with HR9 and Pep-1 by varying the concentrations of the CB1954 prodrug in the presence of NADH as the cofactor.

Conjugate	V_max_ (µMs^−1^)	K_cat_ (s^−1^)	K_m_ (µM)	K_cat_/K_m_ (µM^−1^s^−1^)
NfnB-Cys:HR9	7.98 ± 1.39	9.06 ± 0.82	3443 ± 916	0.00263 ± 4.6 × 10^4^
NfnB-Cys:Pep-1	7.43 ± 1.25	8.44 ± 0.73	2381 ± 695	0.00354 ± 6.5 × 10^4^

**Table 2:** The ratio of the CB1954 hydroxylamine derivatives formed when NfnB-Cys:CPP conjugates were reacted with CB1954 in the presence of NADH as determined by HPLC.

Conjugate	Ratio (2-NHOH:4-NHOH)
NfnB-Cys:HR9	36:64
NfnB-Cys:Pep-1	35:65
